# Supplementary material for: Magneto-Photonic Gene Circuit for Minimally Invasive Control of Gene Expression in Mammalian Cells
Source: ACS Omega. 2026 Mar 5;11(10):16765–74. doi: 10.1021/acsomega.5c13335 (PMC13000775; doi:10.1021/acsomega.5c13335)
Supplement: Supplementary file 1 [file ao5c13335_si_001.pdf]

Supporting Information

Magneto-Photonic Gene Circuit for Minimally Invasive Control of Gene Expression in Mammalian Cells

Enrique Alejandro Castellanos Franco[1], Ryan Radawiec[2], Ashley Slaviero[3], Connor J. Grady[1], Brianna Ricker[4], Galit Pelled[2], Ute Hochgeschwender[3], Taeho Kim[1,5], Assaf A. Gilad[4,5,6,*]

1. Department of Biomedical Engineering, Michigan State University, East Lansing, MI, United States.
2. Department of Mechanical Engineering, Michigan State University, East Lansing, MI, United States.
3. College of Medicine, Central Michigan University, Mount Pleasant, MI, United States.
4. Department of Chemical Engineering and Materials Science, Michigan State University, East Lansing, MI, United States.
5. Institute for Quantitative Health Science and Engineering, Michigan State University, East Lansing, MI, United States.
6. Department of Radiology, Michigan State University, East Lansing, MI, United States.
7. The Scojen Institute for Synthetic Biology, Reichman University, Herzliya, Israel.

* Corresponding author: Assaf Gilad (assaf.a.gilad@gmail.com)

Supplemental Figures

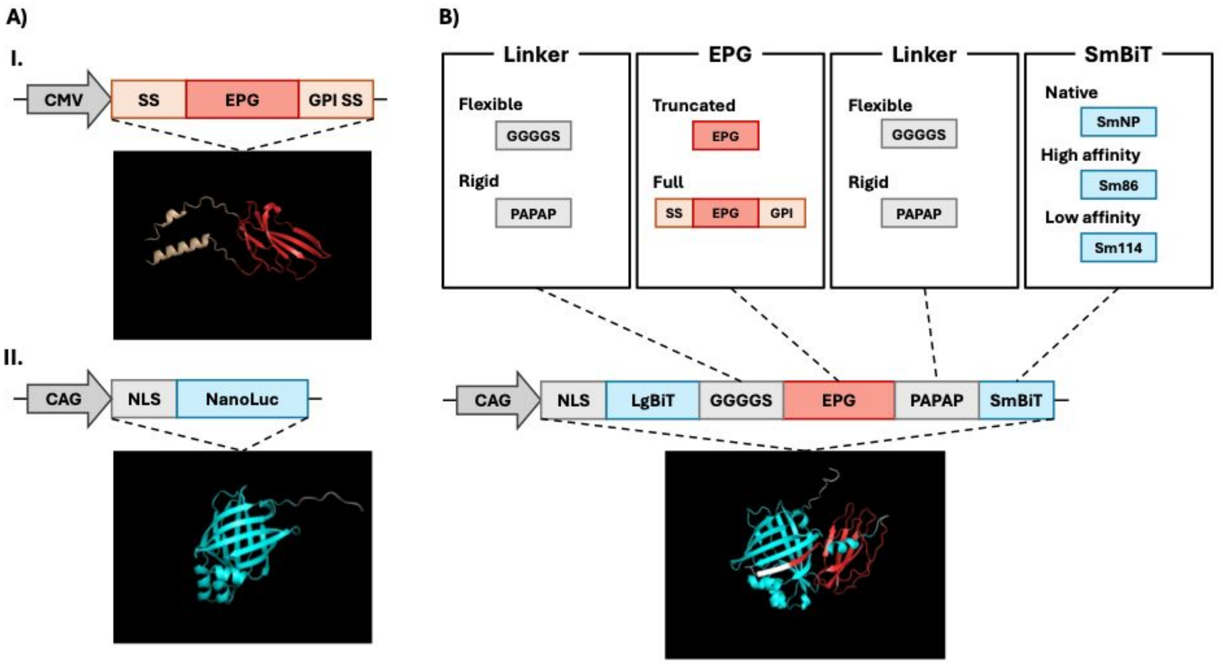

Figure S1: Linear maps and structure prediction models for EPG (A-I), NanoLuc (A-II), and EPG-split-NanoLuc fusion constructs (B). (A-I) The electromagnetic perceptive gene (EPG) protein shares a similar structure to members of the Ly6/uPAR family of proteins, characterized by their three-finger protein domain (red). EPG possesses an N-terminus translocation signal sequence and a C-terminus GPI anchor signal sequence. **(A-II)** NanoLuc luciferase is a luminescent enzyme possessing a b-barrel structure that catalyzes the oxidation of coelenterazine, or its derivatives, producing blue light (460nm) in the process. This version of NanoLuc was fused to an SV40 nuclear localization sequence to improve compatibility with the EL222 system. **(B)** General structure of EPG-NanoLuc fusion constructs. Synthetic fusions were built by fusing split-NanoLuc fragments (large subunit and small subunit) to EPG via 5-amino acid linkers. Each synthetic protein results from the combination of four variable factors: first linker flexibility (flexible or rigid), EPG variant (full or truncated), second linker flexibility (flexible or rigid) and small subunit affinity (native, low or high).

Table S1: Components of EPG-NanoLuc fusion proteins cloned during this study. A total of 24 distinct constructs were prepared and tested, accounting for every possible

combination of the four variable factors. Each protein received a unique identifier that summarizes the specific combination of factors utilized (e.g. fFRNP: full EPG, flexible first linker, rigid second linker, and native variant small subunit).

Construct	First Linker	Magnetoreceptor	Second Linker	SmBiT Variant
**FFNP**	Flexible	Truncated EPG	Flexible	Native peptide
**FRNP**	Flexible	Truncated EPG	Rigid	Native peptide
**RFNP**	Rigid	Truncated EPG	Flexible	Native peptide
**RRNP**	Rigid	Truncated EPG	Rigid	Native peptide
**FF86**	Flexible	Truncated EPG	Flexible	Peptide 86
**FR86**	Flexible	Truncated EPG	Rigid	Peptide 86
**RF86**	Rigid	Truncated EPG	Flexible	Peptide 86
**RR86**	Rigid	Truncated EPG	Rigid	Peptide 86
**FF114**	Flexible	Truncated EPG	Flexible	Peptide 114
**FR114**	Flexible	Truncated EPG	Rigid	Peptide 114
**RF114**	Rigid	Truncated EPG	Flexible	Peptide 114
**RR114**	Rigid	Truncated EPG	Rigid	Peptide 114
**fFFNP**	Flexible	EPG	Flexible	Native peptide
**fFRNP**	Flexible	EPG	Rigid	Native peptide
**fRFNP**	Rigid	EPG	Flexible	Native peptide
**fRRNP**	Rigid	EPG	Rigid	Native peptide
**fFF86**	Flexible	EPG	Flexible	Peptide 86
**fFR86**	Flexible	EPG	Rigid	Peptide 86
**fRF86**	Rigid	EPG	Flexible	Peptide 86
**fRR86**	Rigid	EPG	Rigid	Peptide 86
**fFF114**	Flexible	EPG	Flexible	Peptide 114
**fFR114**	Flexible	EPG	Rigid	Peptide 114
**fRF114**	Rigid	EPG	Flexible	Peptide 114
**fRR114**	Rigid	EPG	Rigid	Peptide 114

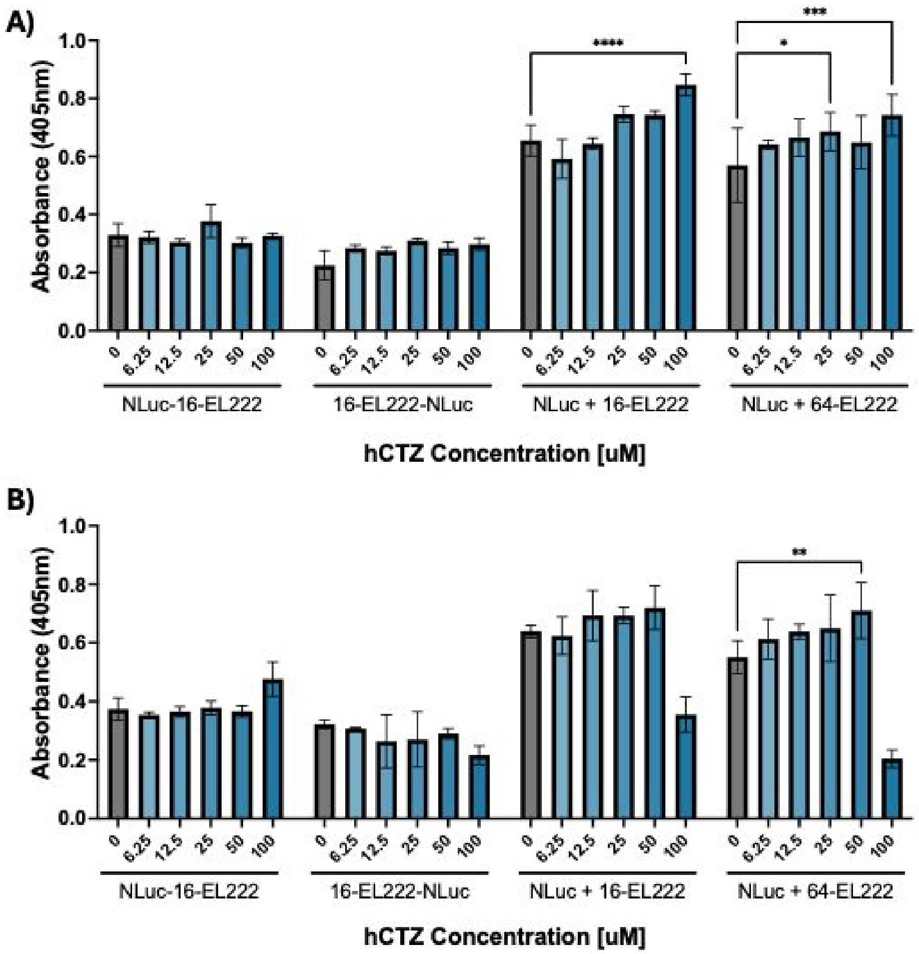

Figure S2: Replicates of substrate concentration experiments for luminescent activation of EL222. (A, B) Replicate assays for quantification of SEAP reporter expression induced by NanoLuc-EL222 fusions or NanoLuc co-expressed with EL222 variants. Statistical significance was calculated at a 5% significance level using Two-way analysis of variance (ANOVA) followed by Dunnett's test. (*) = $P < 0.05$, (**) = $P < 0.01$, (***) = $P < 0.001$, (****) = P = < 0.0001.

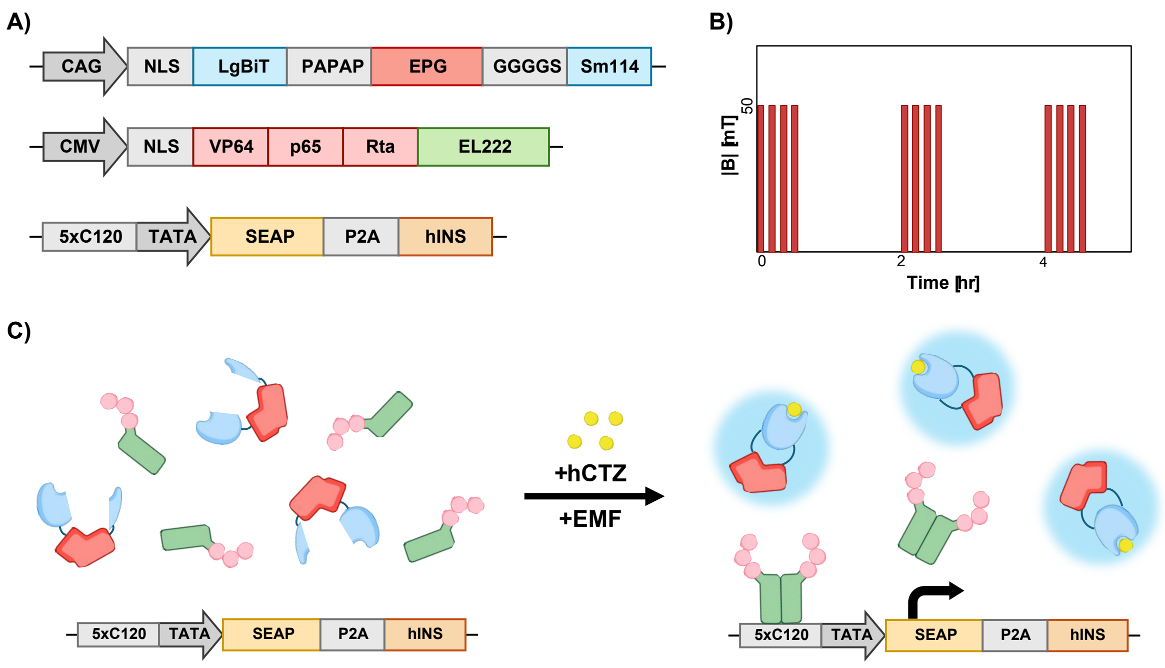

Figure S3: Final design and elements of the magneto-photonic EL222 circuit. (A) Linear map representations of the three circuit components. Nuclear-localized EPG-NanoLuc serves as both an intracellular light source and magneto sensitive component, VPR-EL222 functions as a photo-sensing component and transcription factor, and 5xC120 SEAP acts as inducible reporter for the system. **(B)** Visual representation of the electromagnetic stimulation pattern used in this study. After addition of hCTZ, cells received four magnetic stimulation pulses (50mT) following a 15s-ON 5min-OFF pattern; this is repeated two more times, each separated by a 2-hour interval. **(C)** Illustration of the magneto-photonic circuit during the inactive and active state. Magnetic stimulation induces EPG-driven reconstitution of NanoLuc, which produces blue light in the presence of hCTZ. As a results, EL222 dimerizes, bind DNA and promotes transcription of the reporter.

Sequences of Constructs Used in This Study

VP16 EL222 – Vector: pcDNA3.1 – Promoter: CMV

Start-VP16-EL222-End

ATGGGCCCTAAAAAGAAGCGTAAAGTCGCCCCCCCGACCGATGTCAGCCTGGGG
GACGAGCTCCACTTAGACGGCGAGGACGTGGCGATGGCGCATGCCGACGCGCTA
GACGATTTCGATCTGGACATGTTGGGGGACGGGGATTCCCCGGGCCGGGATTTA
CCCCCCACGACTCCGCCCCCTACGGCGCTCTGGATATGGCCGACTTCGAGTTTGA
GCAGATGTTTACCGATGCCCTTGGAATTGACGAGTACGGTGGGGAATTCGGGGCA
GACGACACACGCGTTGAGGTGCAACCGCCGGCGCAGTGGGTCCTCGACCTGATC
GAGGCCAGCCCGATCGCATCGGTCGTGTCCGATCCGCGTCTCGCCGACAATCCG
CTGATCGCCATCAACCAGGCCTTCACCGACCTGACCGGCTATTCCGAAGAAGAAT
GCGTCGGCCGCAATTGCCGATTCCTGGCAGGTTCCGGCACCGAGCCGTGGCTGA
CCGACAAGATCCGCCAAGGCGTGCGCGAGCACAAGCCGGTGCTGGTCGAGATCC
TGAACTACAAGAAGGACGGCACGCCGTTCCGCAATGCCGTGCTCGTTGCACCGAT
CTACGATGACGACGACGAGCTTCTCTATTTCCTCGGCAGCCAGGTCGAAGTCGAC
GACGACCAGCCCAACATGGGCATGGCGCGCCGCGAACGCGCCGCGGAAATGCTC
AGGACGCTGTCGCCGCGCCAGCTCGAGGTTACGACGCTGGTGGCATCGGGCTTG
CGCAACAAGGAAGTGGCGGCCCGGCTCGGCCTGTCGGAGAAAACCGTCAAGATG
CACCGCGGGCTGGTGATGGAAAAGCTCAACCTGAAGACCAGTGCCGATCTGGTG
CGCATTGCCGTCGAAGCCGGAATCTAA

VP64 EL222 – Vector: pcDNA3.1 – Promoter: CMV

Start-VP64-EL222-End

ATGGGCCCTAAAAAGAAGCGTAAAGTCGACGCATTGGACGATTTTGATCTGGATAT
GCTGGGAAGTGACGCCCTCGATGATTTTGACCTTGACATGCTTGGTTCGGATGCC
CTTGATGACTTTGACCTCGACATGCTCGGCAGTGACGCCCTTGATGATTTCGACCT
GGACATGCTGGAATTCGGGGCAGACGACACACGCGTTGAGGTGCAACCGCCGGC
GCAGTGGGTCCTCGACCTGATCGAGGCCAGCCCGATCGCATCGGTCGTGTCCGA
TCCGCGTCTCGCCGACAATCCGCTGATCGCCATCAACCAGGCCTTCACCGACCTG
ACCGGCTATTCCGAAGAAGAATGCGTCGGCCGCAATTGCCGATTCCTGGCAGGTT
CCGGCACCGAGCCGTGGCTGACCGACAAGATCCGCCAAGGCGTGCGCGAGCACA
AGCCGGTGCTGGTCGAGATCCTGAACTACAAGAAGGACGGCACGCCGTTCCGCA
ATGCCGTGCTCGTTGCACCGATCTACGATGACGACGACGAGCTTCTCTATTTCCTC
GGCAGCCAGGTCGAAGTCGACGACGACCAGCCCAACATGGGCATGGCGCGCCGC
GAACGCGCCGCGGAAATGCTCAGGACGCTGTCGCCGCGCCAGCTCGAGGTTACG
ACGCTGGTGGCATCGGGCTTGCGCAACAAGGAAGTGGCGGCCCGGCTCGGCCTG

TCGGAGAAAACCGTCAAGATGCACCGCGGGCTGGTGATGGAAAAGCTCAACCTGA
AGACCAGTGCCGATCTGGTGCGCATTGCCGTCGAAGCCGGAATCTAA

VPR EL222 – Vector: pcDNA3.1 – Promoter: CMV

Start-VPR-EL222-End

ATGGGCCCTAAAAAGAAGCGTAAAGTCGACGCATTGGACGATTTTGATCTGGATAT
GCTGGGAAGTGACGCCCTCGATGATTTTGACCTTGACATGCTTGGTTCGGATGCC
CTTGATGACTTTGACCTCGACATGCTCGGCAGTGACGCCCTTGATGATTTCGACCT
GGACATGCTGATTAACTCTAGAAGTTCCGGATCTCCGAAAAAGAAACGCAAAGTTG
GTAGCCAGTACCTGCCCGACACCGACGACCGGCACCGGATCGAGGAAAAGCGGA
AGCGGACCTACGAGACATTCAAGAGCATCATGAAGAAGTCCCCCTTCAGCGGCCC
CACCGACCCTAGACCTCCACCTAGAAGAATCGCCGTGCCCAGCAGATCCAGCGCC
AGCGTGCCAAAACCTGCCCCCCAGCCTTACCCCTTCACCAGCAGCCTGAGCACCA
TCAACTACGACGAGTTCCCTACCATGGTGTTCCCCAGCGGCCAGATCTCTCAGGC
CTCTGCTCTGGCTCCAGCCCCTCCTCAGGTGCTGCCTCAGGCTCCTGCTCCTGCA
CCAGCTCCAGCCATGGTGTCTGCACTGGCTCAGGCACCAGCACCCGTGCCTGTGC
TGGCTCCTGGACCTCCACAGGCTGTGGCTCCACCAGCCCCTAAACCTACACAGGC
CGGCGAGGGCACACTGTCTGAAGCTCTGCTGCAGCTGCAGTTCGACGACGAGGA
TCTGGGAGCCCTGCTGGGAAACAGCACCGATCCTGCCGTGTTCACCGACCTGGCC
AGCGTGGACAACAGCGAGTTCCAGCAGCTGCTGAACCAGGGCATCCCTGTGGCC
CCTCACACCACCGAGCCCATGCTGATGGAATACCCCGAGGCCATCACCCGGCTCG
TGACAGGCGCTCAGAGGCCTCCTGATCCAGCTCCTGCCCCTCTGGGAGCACCAG
GCCTGCCTAATGGACTGCTGTCTGGCGACGAGGACTTCAGCTCTATCGCCGATAT
GGATTTCTCAGCCTTGCTGGGCTCTGGCAGCGGCAGCCGGGATTCCAGGGAAGG
GATGTTTTTGCCGAAGCCTGAGGCCGGCTCCGCTATTAGTGACGTGTTTGAGGGC
CGCGAGGTGTGCCAGCCAAAACGAATCCGGCCATTTCATCCTCCAGGAAGTCCAT
GGGCCAACCGCCCACTCCCCGCCAGCCTCGCACCAACACCAACCGGTCCAGTAC
ATGAGCCAGTCGGGTCACTGACCCCGGCACCAGTCCCTCAGCCACTGGATCCAG
CGCCCGCAGTGACTCCCGAGGCCAGTCACCTGTTGGAGGATCCCGATGAAGAGA
CGAGCCAGGCTGTCAAAGCCCTTCGGGAGATGGCCGATACTGTGATTCCCCAGAA
GGAAGAGGCTGCAATCTGTGGCCAAATGGACCTTTCCCATCCGCCCCCAAGGGGC
CATCTGGATGAGCTGACAACCACACTTGAGTCCATGACCGAGGATCTGAACCTGG
ACTCACCCCTGACCCCGGAATTGAACGAGATTCTGGATACCTTCCTGAACGACGA
GTGCCTCTTGCATGCCATGCATATCAGCACAGGACTGTCCATCTTCGACACATCTC
TGTTTGAATTCGGGGCAGACGACACACGCGTTGAGGTGCAACCGCCGGCGCAGT
GGGTCCTCGACCTGATCGAGGCCAGCCCGATCGCATCGGTCGTGTCCGATCCGC
GTCTCGCCGACAATCCGCTGATCGCCATCAACCAGGCCTTCACCGACCTGACCGG
CTATTCCGAAGAAGAATGCGTCGGCCGCAATTGCCGATTCCTGGCAGGTTCCGGC
ACCGAGCCGTGGCTGACCGACAAGATCCGCCAAGGCGTGCGCGAGCACAAGCCG
GTGCTGGTCGAGATCCTGAACTACAAGAAGGACGGCACGCCGTTCCGCAATGCCG

TGCTCGTTGCACCGATCTACGATGACGACGACGAGCTTCTCTATTTCCTCGGCAGC
CAGGTCGAAGTCGACGACGACCAGCCCAACATGGGCATGGCGCGCCGCGAACGC
GCCGCGGAAATGCTCAGGACGCTGTCGCCGCGCCAGCTCGAGGTTACGACGCTG
GTGGCATCGGGCTTGCGCAACAAGGAAGTGGCGGCCCGGCTCGGCCTGTCGGAG
AAAACCGTCAAGATGCACCGCGGGCTGGTGATGGAAAAGCTCAACCTGAAGACCA
GTGCCGATCTGGTGCGCATTGCCGTCGAAGCCGGAATCTAA

5xC120 Firefly – Vector: pcDNA3.1 – Promoter: 5xC120

Start-Firefly-End

ATGGAAGATGCCAAAAACATTAAGAAGGGCCCAGCGCCATTCTACCCACTCGAAG
ACGGGACCGCCGGCGAGCAGCTGCACAAAGCCATGAAGCGCTACGCCCTGGTGC
CCGGCACCATCGCCTTTACCGACGCACATATCGAGGTGGACATTACCTACGCCGA
GTACTTCGAGATGAGCGTTCGGCTGGCAGAAGCTATGAAGCGCTATGGGCTGAAT
ACAAACCATCGGATCGTGGTGTGCAGCGAGAATAGCTTGCAGTTCTTCATGCCCG
TGTTGGGTGCCCTGTTCATCGGTGTGGCTGTGGCCCCAGCTAACGACATCTACAA
CGAGCGCGAGCTGCTGAACAGCATGGGCATCAGCCAGCCCACCGTCGTATTCGT
GAGCAAGAAAGGGCTGCAAAAGATCCTCAACGTGCAAAAGAAGCTACCGATCATA
CAAAAGATCATCATCATGGATAGCAAGACCGACTACCAGGGCTTCCAAAGCATGTA
CACCTTCGTGACTTCCCATTTGCCACCCGGCTTCAACGAGTACGACTTCGTGCCCG
AGAGCTTCGACCGGGACAAAACCATCGCCCTGATCATGAACAGTAGTGGCAGTAC
CGGATTGCCCAAGGGCGTAGCCCTACCGCACCGCACCGCTTGTGTCCGATTCAGT
CATGCCCGCGACCCCATCTTCGGCAACCAGATCATCCCCGACACCGCTATCCTCA
GCGTGGTGCCATTTCACCACGGCTTCGGCATGTTCACCACGCTGGGCTACTTGAT
CTGCGGCTTTCGGGTCGTGCTCATGTACCGCTTCGAGGAGGAGCTATTCTTGCGC
AGCTTGCAAGACTATAAGATTCAATCTGCCCTGCTGGTGCCCACACTATTTAGCTT
CTTCGCTAAGAGCACTCTCATCGACAAGTACGACCTAAGCAACTTGCACGAGATCG
CCAGCGGCGGGGCGCCGCTCAGCAAGGAGGTAGGTGAGGCCGTGGCCAAACGC
TTCCACCTACCAGGCATCCGCCAGGGCTACGGCCTGACAGAAACAACCAGCGCCA
TTCTGATCACCCCCGAAGGGGACGACAAGCCTGGCGCAGTAGGCAAGGTGGTGC
CCTTCTTCGAGGCTAAGGTGGTGGACTTGGACACCGGTAAGACACTGGGTGTGAA
CCAGCGCGGCGAGCTGTGCGTCCGTGGCCCCATGATCATGAGCGGCTACGTTAA
CAACCCCGAGGCTACAAACGCTCTCATCGACAAGGACGGCTGGCTGCACAGCGG
CGACATCGCCTACTGGGACGAGGACGAGCACTTCTTCATCGTGGACCGGCTGAAG
AGCCTGATCAAATACAAGGGCTACCAGGTAGCCCCAGCCGAACTGGAGAGCATCC
TGCTGCAACACCCCAACATCTTCGACGCCGGGGTCGCCGGCCTGCCCGACGACG
ATGCCGGCGAGCTGCCCGCCGCAGTCGTCGTGCTGGAACACGGTAAAACCATGA
CCGAGAAGGAGATCGTGGACTATGTGGCCAGCCAGGTTACAACCGCCAAGAAGCT
GCGCGGTGGTGTTGTGTTCGTGGACGAGGTGCCTAAAGGACTGACCGGCAAGTT

GGACGCCCGCAAGATCCGCGAGATTCTCATTAAGGCCAAGAAGGGCGGCAAGAT
CGCCGTGTAA

5xC120 SEAP-P2A-hINS – Vector: pcDNA3.1 – Promoter: 5xC120

Start-SEAP-P2A-hINS-End

ATGCTGGGGCCCTGCATGCTGCTGCTGCTGCTGCTGGGCCTGAGGCTACAG
CTCTCCCTGGGCATCATCCCAGTTGAGGAGGAGAACCCGGACTTCTGGAACCGCG
AGGCAGCCGAGGCCCTGGGTGCCGCCAAGAAGCTGCAGCCTGCACAGACAGCCG
CCAAGAACCTCATCATCTTCCTGGGCGATGGGATGGGGGTGTCTACGGTGACAGC
TGCCAGGATCCTAAAAGGGCAGAAGAAGGACAAACTGGGGCCTGAGATACCCCTG
GCCATGGACCGCTTCCCATATGTGGCTCTGTCCAAGACATACAATGTAGACAAACA
TGTGCCAGACAGTGGAGCCACAGCCACGGCCTACCTGTGCGGGGTCAAGGGCAA
CTTCCAGACCATTGGCTTGAGTGCAGCCGCCCGCTTTAACCAGTGCAACACGACA
CGCGGCAACGAGGTCATCTCCGTGATGAATCGGGCCAAGAAAGCAGGGAAGTCA
GTGGGAGTGGTAACCACCACACGAGTGCAGCACGCCTCGCCAGCCGGCACCTAC
GCCCACACGGTGAACCGCAACTGGTACTCGGACGCCGACGTGCCTGCCTCGGCC
CGCCAGGAGGGGTGCCAGGACATCGCTACGCAGCTCATCTCCAACATGGACATTG
ACGTGATCCTAGGTGGAGGCCGAAAGTACATGTTTCGCATGGGAACCCCAGACCC
TGAGTACCCAGATGACTACAGCCAAGGTGGGACCAGGCTGGACGGGAAGAATCT
GGTGCAGGAATGGCTGGCGAAGCGCCAGGGTGCCCGGTATGTGTGGAACCGCAC
TGAGCTCATGCAGGCTTCCCTGGACCCGTCTGTGACCCATCTCATGGGTCTCTTTG
AGCCTGGAGACATGAAATACGAGATCCACCGAGACTCCACACTGGACCCCTCCCT
GATGGAGATGACAGAGGCTGCCCTGCGCCTGCTGAGCAGGAACCCCCGCGGCTT
CTTCCTCTTCGTGGAGGGTGGTCGCATCGACCATGGTCATCATGAAAGCAGGGCT
TACCGGGCACTGACTGAGACGATCATGTTCGACGACGCCATTGAGAGGGCGGGC
CAGCTCACCAGCGAGGAGGACACGCTGAGCCTCGTCACTGCCGACCACTCCCAC
GTCTTCTCCTTCGGAGGCTACCCCCTGCGAGGGAGCTCCATCTTCGGGCTGGCCC
CTGGCAAGGCCCGGGACAGGAAGGCCTACACGGTCCTCCTATACGGAAACGGTC
CAGGCTATGTGCTCAAGGACGGCGCCCGGCCGGATGTTACCGAGAGCGAGAGCG
GGAGCCCCGAGTATCGGCAGCAGTCAGCAGTGCCCCTGGACGAAGAGACCCACG
CAGGCGAGGACGTGGCGGTGTTCGCGCGCGGCCCGCAGGCGCACCTGGTTCAC
GGCGTGCAGGAGCAGACCTTCATAGCGCACGTCATGGCCTTCGCCGCCTGCCTG
GAGCCCTACACCGCCTGCGACCTGGCGCCCCCCGCCGGCACCACCGACGCCGC
GCACCCGGGTCGTCGCAAGCGTGGAAGCGGAGCTACTAACTTCAGCCTGCTGAA
GCAGGCTGGAGACGTGGAGGAGAACCCTGGACCTGTCGAATTCATGGCCCTCTG

GATGAGGCTGCTTCCACTTCTTGCGCTCCTGGCGTTGTGGGGACCTGATCCGGCG
GCAGCGTTCGTCAATCAGCATCTGTGCGGGAGTCACCTTGTCGAAGCATTGTACCT
TGTTTGTGGAGAGCGCGGTTTTTTCTATACGCCTAAGACCCGCAGAGAGGCTGAA
GATTTGCAAGTGGGACAAGTGGAGCTTGGAGGAGGGCCGGGTGCCGGTTCCCTC
CAGCCTTTGGCTCTTGAGGGGTCCCTTCAGAAACGCGGGATAGTCGAACAATGTT
GCACAAGCATATGCTCACTTTACCAACTGGAAAATTACTGCAACTAA

NLS NanoLuc – Vector: pcDNA3.1 – Promoter: CAG

Start-NLS-NanoLuc-End

ATGGGCCCTAAAAAGAAGCGTAAAGTCGTCTTCACACTCGAAGATTTCGTTGGGGA
CTGGCGACAGACAGCCGGCTACAACCTGGACCAAGTCCTTGAACAGGGAGGTGT
GTCCAGTTTGTTTCAGAATCTCGGGGTGTCCGTAACTCCGATCCAAAGGATTGTCC
TGAGCGGTGAAAATGGGCTGAAGATCGACATCCATGTCATCATCCCGTATGAAGG
TCTGAGCGGCGACCAAATGGGCCAGATCGAAAAAATTTTTAAGGTGGTGTACCCT
GTGGATGATCATCACTTTAAGGTGATCCTGCACTATGGCACACTGGTAATCGACGG
GGTTACGCCGAACATGATCGACTATTTCGGACGGCCGTATGAAGGCATCGCCGTG
TTCGACGGCAAAAAGATCACTGTAACAGGGACCCTGTGGAACGGCAACAAAATTAT
CGACGAGCGCCTGATCAACCCCGACGGCTCCCTGCTGTTCCGAGTAACCATCAAC
GGAGTGACCGGCTGGCGGCTGTGCGAACGCATTCTGGCGTAA

EPG-NanoLuc FFNP – Vector: pcDNA3.1 – Promoter: CAG

Start-NLS-LgBiT-Linker-EPG-Linker-SmBiT-End

ATGGGCCCTAAAAAGAAGCGTAAAGTCGTCTTCACACTCGAAGATTTCGTTGGGGA
CTGGGAACAGACAGCCGCCTACAACCTGGACCAAGTCCTTGAACAGGGAGGTGTG
TCCAGTTTGCTGCAGAATCTCGCCGTGTCCGTAACTCCGATCCAAAGGATTGTCCG
GAGCGGTGAAAATGCCCTGAAGATCGACATCCATGTCATCATCCCGTATGAAGGT
CTGAGCGCCGACCAAATGGCCCAGATCGAAGAGGTGTTTAAGGTGGTGTACCCTG
TGGATGATCATCACTTTAAGGTGATCCTGCCCTATGGCACACTGGTAATCGACGGG
GTTACGCCGAACATGCTGAACTATTTCGGACGGCCGTATGAAGGCATCGCCGTGT
TCGACGGCAAAAAGATCACTGTAACAGGGACCCTGTGGAACGGCAACAAAATTAT
CGACGAGCGCCTGATCACCCCCGACGGCTCCATGCTGTTCCGAGTAACCATCAAC
GGAGGAGGCGGTAGTCTTACCTGTAACACATGCTCAGTGAGTCTGATTGGAATAT
GTCTGAATCCCGCAACAGCGACTTGCTCCACCAACACATCCGTCTGCACCACAGG
AAGAGCCAGTTTCACGGGCGTCCTCGGCTTCCTGGGCTTCAACTCCCAGGGCTGC
ACGGAGGGAGCTCAGTGTAATGGCACCGTGTCCGGGTCCATCCTGGGTGCGTCG

TACACGGTCACTCAAACCTGCTGCAGCACAAACAACTGCAACCCCGTGACCAGCG
GCGCCTCCGGAGGAGGCGGCTCCGTGACCGGCTGGCGGCTGTGCGAACGCATTC
TGGCGTAA

EPG-NanoLuc FR86 – Vector: pcDNA3.1 – Promoter: CAG

Start-NLS-LgBiT-Linker-EPG-Linker-SmBiT-End

ATGGGCCCTAAAAAGAAGCGTAAAGTCGTCTTCACACTCGAAGATTTCGTTGGGGA
CTGGGAACAGACAGCCGCCTACAACCTGGACCAAGTCCTTGAACAGGGAGGTGTG
TCCAGTTTGCTGCAGAATCTCGCCGTGTCCGTAACTCCGATCCAAAGGATTGTCCG
GAGCGGTGAAAATGCCCTGAAGATCGACATCCATGTCATCATCCCGTATGAAGGT
CTGAGCGCCGACCAAATGGCCCAGATCGAAGAGGTGTTTAAGGTGGTGTACCCTG
TGGATGATCATCACTTTAAGGTGATCCTGCCCTATGGCACACTGGTAATCGACGGG
GTTACGCCGAACATGCTGAACTATTTCGGACGGCCGTATGAAGGCATCGCCGTGT
TCGACGGCAAAAAGATCACTGTAACAGGGACCCTGTGGAACGGCAACAAAATTAT
CGACGAGCGCCTGATCACCCCCGACGGCTCCATGCTGTTCCGAGTAACCATCAAC
GGAGGAGGCGGTAGTCTTACCTGTAACACATGCTCAGTGAGTCTGATTGGAATAT
GTCTGAATCCCGCAACAGCGACTTGCTCCACCAACACATCCGTCTGCACCACAGG
AAGAGCCAGTTTCACGGGCGTCCTCGGCTTCCTGGGCTTCAACTCCCAGGGCTGC
ACGGAGGGAGCTCAGTGTAATGGCACCGTGTCCGGGTCCATCCTGGGTGCGTCG
TACACGGTCACTCAAACCTGCTGCAGCACAAACAACTGCAACCCCGTGACCAGCG
GCGCCTCCCCTGCCCCAGCTCCCGTGTCCGGCTGGCGGCTGTTCAAGAAAATTTC
TTAA

EPG-NanoLuc RF86 – Vector: pcDNA3.1 – Promoter: CAG

Start-NLS-LgBiT-Linker-EPG-Linker-SmBiT-End

ATGGGCCCTAAAAAGAAGCGTAAAGTCGTCTTCACACTCGAAGATTTCGTTGGGGA
CTGGGAACAGACAGCCGCCTACAACCTGGACCAAGTCCTTGAACAGGGAGGTGTG
TCCAGTTTGCTGCAGAATCTCGCCGTGTCCGTAACTCCGATCCAAAGGATTGTCCG
GAGCGGTGAAAATGCCCTGAAGATCGACATCCATGTCATCATCCCGTATGAAGGT
CTGAGCGCCGACCAAATGGCCCAGATCGAAGAGGTGTTTAAGGTGGTGTACCCTG
TGGATGATCATCACTTTAAGGTGATCCTGCCCTATGGCACACTGGTAATCGACGGG
GTTACGCCGAACATGCTGAACTATTTCGGACGGCCGTATGAAGGCATCGCCGTGT
TCGACGGCAAAAAGATCACTGTAACAGGGACCCTGTGGAACGGCAACAAAATTAT
CGACGAGCGCCTGATCACCCCCGACGGCTCCATGCTGTTCCGAGTAACCATCAAC
CCTGCCCCAGCTCCCCTTACCTGTAACACATGCTCAGTGAGTCTGATTGGAATATG
TCTGAATCCCGCAACAGCGACTTGCTCCACCAACACATCCGTCTGCACCACAGGA

AGAGCCAGTTTCACGGGCGTCCTCGGCTTCCTGGGCTTCAACTCCCAGGGCTGCA
CGGAGGGAGCTCAGTGTAATGGCACCGTGTCCGGGTCCATCCTGGGTGCGTCGT
ACACGGTCACTCAAACCTGCTGCAGCACAAACAACTGCAACCCCGTGACCAGCGG
CGCCTCCGGAGGAGGCGGCTCCGTGTCCGGCTGGCGGCTGTTCAAGAAAATTTCT
TAA

EPG-NanoLuc RF114 – Vector: pcDNA3.1 – Promoter: CAG

Start-NLS-LgBiT-Linker-EPG-Linker-SmBiT-End

ATGGGCCCTAAAAAGAAGCGTAAAGTCGTCTTCACACTCGAAGATTTCGTTGGGGA
CTGGGAACAGACAGCCGCCTACAACCTGGACCAAGTCCTTGAACAGGGAGGTGTG
TCCAGTTTGCTGCAGAATCTCGCCGTGTCCGTAACTCCGATCCAAAGGATTGTCCG
GAGCGGTGAAAATGCCCTGAAGATCGACATCCATGTCATCATCCCGTATGAAGGT
CTGAGCGCCGACCAAATGGCCCAGATCGAAGAGGTGTTTAAGGTGGTGTACCCTG
TGGATGATCATCACTTTAAGGTGATCCTGCCCTATGGCACACTGGTAATCGACGGG
GTTACGCCGAACATGCTGAACTATTTCGGACGGCCGTATGAAGGCATCGCCGTGT
TCGACGGCAAAAAGATCACTGTAACAGGGACCCTGTGGAACGGCAACAAAATTAT
CGACGAGCGCCTGATCACCCCCGACGGCTCCATGCTGTTCCGAGTAACCATCAAC
CCTGCCCCAGCTCCCCTTACCTGTAACACATGCTCAGTGAGTCTGATTGGAATATG
TCTGAATCCCGCAACAGCGACTTGCTCCACCAACACATCCGTCTGCACCACAGGA
AGAGCCAGTTTCACGGGCGTCCTCGGCTTCCTGGGCTTCAACTCCCAGGGCTGCA
CGGAGGGAGCTCAGTGTAATGGCACCGTGTCCGGGTCCATCCTGGGTGCGTCGT
ACACGGTCACTCAAACCTGCTGCAGCACAAACAACTGCAACCCCGTGACCAGCGG
CGCCTCCGGAGGAGGCGGCTCCGTGACGGGATACAGACTGTTCGAGGAGATCCT
TTAA

EPG-NanoLuc fFFNP – Vector: pcDNA3.1 – Promoter: CAG

Start-NLS-LgBiT-Linker-EPG-Linker-SmBiT-End

ATGGGCCCTAAAAAGAAGCGTAAAGTCGTCTTCACACTCGAAGATTTCGTTGGGGA
CTGGGAACAGACAGCCGCCTACAACCTGGACCAAGTCCTTGAACAGGGAGGTGTG
TCCAGTTTGCTGCAGAATCTCGCCGTGTCCGTAACTCCGATCCAAAGGATTGTCCG
GAGCGGTGAAAATGCCCTGAAGATCGACATCCATGTCATCATCCCGTATGAAGGT
CTGAGCGCCGACCAAATGGCCCAGATCGAAGAGGTGTTTAAGGTGGTGTACCCTG
TGGATGATCATCACTTTAAGGTGATCCTGCCCTATGGCACACTGGTAATCGACGGG
GTTACGCCGAACATGCTGAACTATTTCGGACGGCCGTATGAAGGCATCGCCGTGT
TCGACGGCAAAAAGATCACTGTAACAGGGACCCTGTGGAACGGCAACAAAATTAT
CGACGAGCGCCTGATCACCCCCGACGGCTCCATGCTGTTCCGAGTAACCATCAAC

GGAGGAGGCGGTAGTAAGTGTGTACTTTTGGGATTCGCAGCAGTGATCGGATTCT
TCGCGATCGCGGAGTCTCTTACCTGTAACACATGCTCAGTGAGTCTGATTGGAATA
TGTCTGAATCCCGCAACAGCGACTTGCTCCACCAACACATCCGTCTGCACCACAG
GAAGAGCCAGTTTCACGGGCGTCCTCGGCTTCCTGGGCTTCAACTCCCAGGGCTG
CACGGAGGGAGCTCAGTGTAATGGCACCGTGTCCGGGTCCATCCTGGGTGCGTC
GTACACGGTCACTCAAACCTGCTGCAGCACAAACAACTGCAACCCCGTGACCAGC
GGCGCCTCCTACGTCCAGATCTCCGTCAGCGCGGCCCTGAGCGCCGCCCTGCTG
GCCTGCGTCTGGGGCCAGTCCGTCTACGGAGGAGGCGGCTCCGTGACCGGCTGG
CGGCTGTGCGAACGCATTCTGGCGTAA

EPG-NanoLuc fFF86 – Vector: pcDNA3.1 – Promoter: CAG

Start-NLS-LgBiT-Linker-EPG-Linker-SmBiT-End

ATGGGCCCTAAAAAGAAGCGTAAAGTCGTCTTCACACTCGAAGATTTCGTTGGGGA
CTGGGAACAGACAGCCGCCTACAACCTGGACCAAGTCCTTGAACAGGGAGGTGTG
TCCAGTTTGCTGCAGAATCTCGCCGTGTCCGTAACTCCGATCCAAAGGATTGTCCG
GAGCGGTGAAAATGCCCTGAAGATCGACATCCATGTCATCATCCCGTATGAAGGT
CTGAGCGCCGACCAAATGGCCCAGATCGAAGAGGTGTTTAAGGTGGTGTACCCTG
TGGATGATCATCACTTTAAGGTGATCCTGCCCTATGGCACACTGGTAATCGACGGG
GTTACGCCGAACATGCTGAACTATTTCGGACGGCCGTATGAAGGCATCGCCGTGT
TCGACGGCAAAAAGATCACTGTAACAGGGACCCTGTGGAACGGCAACAAAATTAT
CGACGAGCGCCTGATCACCCCCGACGGCTCCATGCTGTTCCGAGTAACCATCAAC
GGAGGAGGCGGTAGTAAGTGTGTACTTTTGGGATTCGCAGCAGTGATCGGATTCT
TCGCGATCGCGGAGTCTCTTACCTGTAACACATGCTCAGTGAGTCTGATTGGAATA
TGTCTGAATCCCGCAACAGCGACTTGCTCCACCAACACATCCGTCTGCACCACAG
GAAGAGCCAGTTTCACGGGCGTCCTCGGCTTCCTGGGCTTCAACTCCCAGGGCTG
CACGGAGGGAGCTCAGTGTAATGGCACCGTGTCCGGGTCCATCCTGGGTGCGTC
GTACACGGTCACTCAAACCTGCTGCAGCACAAACAACTGCAACCCCGTGACCAGC
GGCGCCTCCTACGTCCAGATCTCCGTCAGCGCGGCCCTGAGCGCCGCCCTGCTG
GCCTGCGTCTGGGGCCAGTCCGTCTACGGAGGAGGCGGCTCCGTGTCCGGCTGG
CGGCTGTTCAAGAAAATTTCTTAA

EPG-NanoLuc fFR86 – Vector: pcDNA3.1 – Promoter: CAG

Start-NLS-LgBiT-Linker-EPG-Linker-SmBiT-End

ATGGGCCCTAAAAAGAAGCGTAAAGTCGTCTTCACACTCGAAGATTTCGTTGGGGA
CTGGGAACAGACAGCCGCCTACAACCTGGACCAAGTCCTTGAACAGGGAGGTGTG
TCCAGTTTGCTGCAGAATCTCGCCGTGTCCGTAACTCCGATCCAAAGGATTGTCCG

GAGCGGTGAAAATGCCCTGAAGATCGACATCCATGTCATCATCCCGTATGAAGGT
CTGAGCGCCGACCAAATGGCCCAGATCGAAGAGGTGTTTAAGGTGGTGTACCCTG
TGGATGATCATCACTTTAAGGTGATCCTGCCCTATGGCACACTGGTAATCGACGGG
GTTACGCCGAACATGCTGAACTATTTCGGACGGCCGTATGAAGGCATCGCCGTGT
TCGACGGCAAAAAGATCACTGTAACAGGGACCCTGTGGAACGGCAACAAAATTAT
CGACGAGCGCCTGATCACCCCCGACGGCTCCATGCTGTTCCGAGTAACCATCAAC
GGAGGAGGCGGTAGTAAGTGTGTACTTTTGGGATTCGCAGCAGTGATCGGATTCT
TCGCGATCGCGGAGTCTCTTACCTGTAACACATGCTCAGTGAGTCTGATTGGAATA
TGTCTGAATCCCGCAACAGCGACTTGCTCCACCAACACATCCGTCTGCACCACAG
GAAGAGCCAGTTTCACGGGCGTCCTCGGCTTCCTGGGCTTCAACTCCCAGGGCTG
CACGGAGGGAGCTCAGTGTAATGGCACCGTGTCCGGGTCCATCCTGGGTGCGTC
GTACACGGTCACTCAAACCTGCTGCAGCACAAACAACTGCAACCCCGTGACCAGC
GGCGCCTCCTACGTCCAGATCTCCGTCAGCGCGGCCCTGAGCGCCGCCCTGCTG
GCCTGCGTCTGGGGCCAGTCCGTCTACCCTGCCCCAGCTCCCGTGTCCGGCTGG
CGGCTGTTCAAGAAAATTTCTTAA

EPG-NanoLuc fRR114 – Vector: pcDNA3.1 – Promoter: CAG

Start-NLS-LgBiT-Linker-EPG-Linker-SmBiT-End

ATGGGCCCTAAAAAGAAGCGTAAAGTCGTCTTCACACTCGAAGATTTCGTTGGGGA
CTGGGAACAGACAGCCGCCTACAACCTGGACCAAGTCCTTGAACAGGGAGGTGTG
TCCAGTTTGCTGCAGAATCTCGCCGTGTCCGTAACTCCGATCCAAAGGATTGTCCG
GAGCGGTGAAAATGCCCTGAAGATCGACATCCATGTCATCATCCCGTATGAAGGT
CTGAGCGCCGACCAAATGGCCCAGATCGAAGAGGTGTTTAAGGTGGTGTACCCTG
TGGATGATCATCACTTTAAGGTGATCCTGCCCTATGGCACACTGGTAATCGACGGG
GTTACGCCGAACATGCTGAACTATTTCGGACGGCCGTATGAAGGCATCGCCGTGT
TCGACGGCAAAAAGATCACTGTAACAGGGACCCTGTGGAACGGCAACAAAATTAT
CGACGAGCGCCTGATCACCCCCGACGGCTCCATGCTGTTCCGAGTAACCATCAAC
CCTGCCCCAGCTCCCAAGTGTGTACTTTTGGGATTCGCAGCAGTGATCGGATTCTT
CGCGATCGCGGAGTCTCTTACCTGTAACACATGCTCAGTGAGTCTGATTGGAATAT
GTCTGAATCCCGCAACAGCGACTTGCTCCACCAACACATCCGTCTGCACCACAGG
AAGAGCCAGTTTCACGGGCGTCCTCGGCTTCCTGGGCTTCAACTCCCAGGGCTGC
ACGGAGGGAGCTCAGTGTAATGGCACCGTGTCCGGGTCCATCCTGGGTGCGTCG
TACACGGTCACTCAAACCTGCTGCAGCACAAACAACTGCAACCCCGTGACCAGCG
GCGCCTCCTACGTCCAGATCTCCGTCAGCGCGGCCCTGAGCGCCGCCCTGCTGG
CCTGCGTCTGGGGCCAGTCCGTCTACCCTGCCCCAGCTCCCGTGACGGGATACA
GACTGTTCGAGGAGATCCTTTAA
